# Dexamethasone as an adjuvant to continuous erector spinae plane block for postoperative analgesia after video-assisted thoracoscopic surgery for pulmonary nodule surgery: a randomized controlled trial

**DOI:** 10.1080/07853890.2026.2625547

**Published:** 2026-02-12

**Authors:** Liang Fang, Haolin Zhang, Jia Nie, Yiyong Wei, Huanhuan Ma, Peng Lu, Yu Zhang, Wei Chen, Haiying Wang

**Affiliations:** a Department of Anesthesiology, Affiliated Hospital of Zunyi Medical University, Zunyi, Guizhou, China; b Key laboratory of anesthesia and organ protection, Zunyi Medical College, Guizhou, China; c Department of Anaesthesiology, Army Medical University Xinqiao Hospital, Chongqing, China; d Department of Anesthesiology, Longgang District Maternity and Child Healthcare Hospital of Shenzhen City (Longgang Maternity and Child Institute of Shantou University Medical College), Shenzhen, Guangdong, China

**Keywords:** Catheter, dexamethasone, erector spinae plane block, postoperative pain, quality of recovery, video-assisted thoracoscopic surgery

## Abstract

**Background:**

While dexamethasone is proven to enhance single-shot erector spinae plane block (ESPB), its role as an adjuvant in continuous ESPB catheters is unclear. This randomised controlled trial evaluated whether adding dexamethasone to ropivacaine improves analgesia after video-assisted thoracoscopic surgery (VATS).

**Methods:**

85 patients undergoing VATS with continuous ESPB were randomised to receive postoperative infusion of either 0.2% ropivacaine(C-ESPB group) or ropivacaine with 10 mg dexamethasone(D + C-ESPB group). The primary outcome was resting pain visual analog scale (VAS)at 12 h postoperatively, while secondary outcomes included QoR-15 scores, tramadol consumption, time to first analgesic requirement, postoperative adverse events, 3-month incidence of chronic pain, catheter-related complications, pain intensity at other times, and hospital stay.

**Results:**

The D + C-ESPB group had significantly lower resting pain at 12 h [2.56 (1.03) vs 3.24 (1.21), mean difference −0.680, *p* = 0.006]; and lower coughing pain at 12 h [4.60 (1.48) vs 5.69 (1.35), mean difference 1.086, *p* < 0.001], with analgesic superiority sustained through 72 h. Quality of Recovery-15 scores were higher at 12 h [124.70 (12.48) vs 117.26 (12.24); mean difference −7.436, *p* = 0.007] and 48 h [141.60 (5.51) vs 138.98 (6.64); mean difference −2.628, *p* = 0.050]; Total tramadol consumption over 72 h was markedly reduce [0 (0,100) vs 100 (75,100), *z* = –3.807, *p* < 0.001], and hospital stay was shorter [Mean (SD) 6.09 (1.34) d vs 6.93 (1.55)d, *p* < 0.001]. The intervention did not, however, alter the 3-month incidence of chronic postsurgical pain (31% vs 34%, *p* = 0.756).

**Conclusion:**

Dexamethasone significantly enhances the analgesic efficacy of continuous ESPB, improving early pain control, recovery quality, and opioid-sparing after VATS, but does not reduce the incidence of chronic persistent surgical pain.

## KEY MESSAGE

This was a forward-looking randomized controlled trial.

Dexamethasone, when given as an adjuvant, improved the pain relief provided by continuous ESPB, which in turn improved the quality of postoperative recovery and supported early rehabilitation for patients having thoracoscopic lung nodule resection.

Continuous ESPB has the potential to be a useful strategy for managing pain and protecting organs in patients undergoing thoracoscopic surgery for lung nodules.

## 1. Introduction

Video-assisted thoracoscopic surgery (VATS) is the standard approach for pulmonary nodule resection. Despite its minimally invasive nature, moderate to severe postoperative pain remains prevalent, not only delaying recovery but also increasing the risk of chronic postoperative pain (CPSP) [1,2]. Consequently, optimising analgesic strategies is crucial for accelerating recovery following thoracic surgery [3].

The PROSPECT guidelines recommend regional anaesthetic techniques such as the erector spinae plane block (ESPB) as the core of multimodal analgesia [4]. Studies indicate that while a single ESPB injection provides effective early analgesia, its duration of action is approximately 12 h [5,6], insufficient to cover the entire acute pain phase. Theoretically, catheter use may prolong analgesia duration; however, the quality of continuous ESPB analgesia may fluctuate and prove inadequate at times [7–9]. This inconsistency stems from the uncertain mechanism of action of this technique and the unpredictability of drug diffusion [9]. In contrast, dexamethasone consistently enhances the intensity and duration of single-injection peripheral nerve blocks [10–12]. However, the synergistic effects of dexamethasone with continuous local anaesthetic infusion in ESPBs remain unexamined in rigorous studies. Does dexamethasone merely prolong anaesthetic effects or fundamentally enhance the reliability and density of continuous blocks? This unresolved question limits our ability to optimise this promising minimally invasive analgesic technique.

Selecting early postoperative resting pain as the primary endpoint holds strategic significance. Both continuous ESPB and dexamethasone independently prolong analgesia. Intraoperative systemic medications have passed their peak effect by 12 h postoperatively, and the efficacy of single-block techniques begins to wane. Assessing resting pain at 12 h postoperatively allows precise evaluation of the sustained synergistic effect of the combined regimen. Superior early pain control also represents the most direct pathway to reducing opioid requirements and potentially altering the trajectory of chronic pain development [13,14].

Consequently, this study aims to validate an innovative protocol integrating dexamethasone into continuous ESPB infusion. The primary objective is to evaluate its impact on resting pain at 12 h postoperatively. We hypothesize that dexamethasone will produce more potent and sustained analgesia, leading to opioid savings and improved incidence of chronic pain at 3 months post-surgery.

## 2. Methods and materials

This is a single-center, randomized, controlled trial approved by the Ethics Committee of the Army Medical University Xinqiao Hospital (ID: 2022-Research No. 293-01; June 15, 2022). Written informed consent was acquired from all participants. The trial was registered with the China Clinical Trial Registry before commencement (Registration No. ChiCTR2200066531; Registration Date: December 8, 2022; URL: http://www.chictr.org.cn/listbycreater.aspx). This study conforms to the Consolidated Standards of Reporting Trials (CONSORT) and the CONSORT extension for trials reporting patient-related outcomes [15,16]. The study ran from December 10, 2022, to August 30, 2023, and no modifications were made to the protocol after the start of the trial. This differs from the planned recruitment period (8 December 2022 to 31 May 2023) pre-registered on the China Clinical Trial Centre website. This discrepancy arose because the anticipated early phase of the study coincided with the initial stages of China’s COVID-19 reopening, prompting concerns that the virus might increase patient complications. Consequently, we delayed subject screening by one month. We confirm this adjustment did not alter the study’s original protocol, inclusion/exclusion criteria, or primary endpoints. All analyses are based on the cohort of patients actually enrolled.

*Inclusion criteria:* 1) male and female patients aged 18–70 years; 2) American Society of Anesthesiologists(ASA) physical status I to III; 3) body mass index(BMI) 18–30 kg/m^2^; 4) patients scheduled to undergo single-port or multi-port video-assisted thoracoscopic lung nodule resection; 5) patients requiring postoperative analgesia; 6) voluntary participation and signed informed consent.

*Exclusion criteria:* 1) contraindications to regional blockade; 2) puncture site or systemic infection; 3) previous or current neurological deficits; 4) surgical history, trauma, or chronic pain at the puncture site; 5) long-term corticosteroid use or allergies, including local anesthetic allergies; 6) expected postoperative poor pulmonary function requiring mechanical ventilation; 7) conversion to open thoracotomy or significant intraoperative bleeding during VATS; 8) patients with cardiac diseases and liver or kidney function abnormalities; 9) history of opioid abuse, alcoholism, or long-term NSAID use preoperatively; 10) patients unable to communicate or cooperate; 11) participation in other clinical trials or use of experimental drugs within 3 months before study enrollment; 12) any reason deemed inappropriate for inclusion by the researchers.

### 2.1. Randomization, blinding

Randomization was performed by the randomized control investigator according to random numbers on a 1:1 basis. The randomization number was given by a statistician in a sealed opaque envelope to the nursing staff, who configured the analgesic pumps in a separate room where the pumps were dispensed, and the nursing staff determined the analgesic regimen based on the randomization number.

Divided into a test group D + C-ESPB group and a control group C-ESPB group according to the postoperative analgesic regimen, and Members of the surgical, anesthetic, Acute Pain Service (APS), researcher, and nursing staff were blinded to the intervention, as well as the patient. To ensure blinding: (1)the caregiver who configured the analgesic pump was different from the caregiver who managed the patient intraoperatively and postoperatively;(2) the pain pumps were wrapped in opaque bags from the start of the infusion to the end of the infusion.

### 2.2. Regional block

All patients were admitted to the anesthesia preparation room 60 min before induction of general anesthesia to complete the regional block. All patients were given standard monitoring and mask oxygen (15 L/min) after admission to the room, intravenous access was established, and the lateral position was changed (with the affected side on top) for sedation and analgesia (propofol infusion of 2 mg/kg-h + sufentanil 5-10μg), and spontaneous respiration was successfully preserved. or convex array probe (10–12MHz, Shenzhen Huasheng Medical Co. Ltd.), Shenzhen Huasheng Medical Co. Ltd.) was implemented to implement regional blockade corresponding to the subgroups. Corresponding regional blocks were implemented as follows: all patients in the two groups took the lateral position (with the affected side on top), and firstly touched to determine the C7 bony landmarks and marked them, and then sequentially searched for and determined the spinous processes above and below the proposed puncture segments caudally, and localized the T4–5 intervertebral space. Ultrasound images of the spinous processes and transverse processes were obtained by first sweeping cross-sectionally at the level of T5, and after identifying the transverse processes of the segment to be blocked, the probe was rotated by 90 degrees, and the long axis was swept again to localize the spinal processes, including the tip of the transverse processes and the three layers of muscle above them. The high-frequency linear probe was placed along the long axis approximately 3 cm adjacent to the T6 spinous process, and three layers of muscle were visible on the bony acoustic surface of the transverse process, namely the trapezius, rhomboid, and erector spinae muscles, from superficial to deep, respectively, and the block needle was entered from the caudal/cephalad end of the patient with an 18 G Peripheral Plexus Stimulation Kit (Contiplex^®^D, B. Braun Medical) in-plane puncture technique, and the tip of the needle was pressed against the transverse process to The tip of the needle was placed against the lateral aspect of the transverse process, i.e. the tip of the transverse process, and withdrawn without blood, and 30 ml of 0.4% ropivacaine hydrochloride injection (Reg. No. H20140763, AstraZeneca AB) was injected into the deep surface of the erector spinae muscle. Care was taken at the beginning of the injection that the injection should not be injected into the muscle but rather into the fascial space between the transverse process and the erector spinae muscle. In addition, the separation of the erector spinae muscle from the surface of the transverse process is a good sign, suggesting that the local anesthetic was injected at the correct site. The deep surface of the erector spinae muscle was pushed away, and the presence of a hypoechoic liquid dark area on the surface of the transverse process was a sign of a successful block. The 18 G cannula was then secured to withdraw the 20 G puncture needle, and a 20 G nylon epidural catheter (Perifix^®^ PinPad) was placed 3–5 cm in length. 2 mL of 0.9% sodium chloride solution was pushed in to ensure that the tip of the catheter was in place, and then the catheter was attached to a luer-lock connector with antimicrobial filters and secured to the patient’s back with a surgical sterile dressing. The extent of the block was tested and determined using the pinprick method 30 min after the operation.

During the procedure, if the patient’s heart rate dropped below 45 bpm, atropine 0.3–0.5 mg was intravenously injected. If the SBP dropped more than 30% below baseline or 90 mmHg, 6 mg ephedrine was administered intravenously. If the SBP increased more than 30% above baseline, urapidil 5–12.5 mg was intravenously injected, with repeated doses if necessary. If regional blockade or catheter placement failed or systemic toxicity of local anesthetics occurred, the study was immediately halted, and the patient was excluded from the study. In cases of systemic toxicity of local anesthetics, standard resuscitative measures for local anesthetic toxicity were followed.

### 2.3. Anesthesia and analgesia

All enrolled patients were provided standardized monitoring upon entering the operating room and underwent double-lumen endotracheal tube general anesthesia. Induction was achieved with midazolam 0.1 mg/kg, propofol 1.5 mg/kg, rocuronium 0.6 mg/kg, and sufentanil 0.2–0.5 μg/kg. After successful induction, double-lumen endotracheal tube placement (37 F for males, 35 F for females) was confirmed with a bronchoscope. Anesthesia was maintained with sevoflurane (1–3%) and propofol (4–12 mg/kg/h), keeping the BIS index between 40 and 60. Rocuronium 0.2 mg/kg intravenous injection or remifentanil (0.2–0.4 μg/kg/min) infusion was administered as needed to control mean arterial pressure (MAP) and heart rate within ±20% of baseline (ephedrine was used if necessary). Single-lung ventilation was initiated 10 min before the surgery. Ventilation parameters included a fraction of inspired oxygen (FiO_2_) of 100% (reduced to maintain SpO_2_ at least >95% after stabilizing single-lung ventilation), tidal volume (TV) of 4–6ml/kg, respiratory rate of 14–18 breaths/min, positive end-expiratory pressure (PEEP) of 4–6 cmH_2_O, and end-tidal CO_2_ (*P*_E_TCO_2_) of 35–45 mmHg. Manual ventilation was used to assess the full re-expansion of the collapsed lung before switching to bilateral ventilation. 10 min before the end of the surgery, all patients received an intravenous injection of 5 mg ondansetron and 50 mg flurbiprofen. After surgery, the double-lumen endotracheal tube was replaced, and the patient was transferred to the Post-Anesthesia Care Unit (PACU). In the PACU, the staff connected patients to corresponding analgesic pumps (containing 300 ml of solution with or without 10 mg dexamethasone) housed in opaque bags for continuous ESPB (parameters: initial dose of 10 ml, redosing every 3 h with 15 ml, patient-controlled dose of 5 ml, lockout interval of 30 min). If the analgesic pump solution was insufficient for 72 h of pain relief, the same solution was prepared again for continued analgesia. Postoperatively, in addition to the continuous ESPB analgesia pump, patients who were not yet able to eat orally were given 50 mg of flurbiprofen intravenously every 12 h. Once oral intake was resumed, patients were given oral lornoxicam slow-release tablets (2 tablets/12 h). Continuous ESPB analgesia was maintained until one hour before patient discharge. All patients had 3 mL of peripheral venous blood collected preoperatively and 48 h postoperatively into sodium heparin anticoagulant tubes, which were left at room temperature for 30 min, then centrifuged at 3000 rpm and 4 °C for 10 min. The supernatant (plasma) was stored at −80 °C. TNF-α, IL-6, and IL-8 levels in the plasma were measured using ELISA.

During surgery, if the patient’s heart rate fell below 45 bpm, atropine 0.3–0.5 mg was injected intravenously. If the SBP dropped more than 30% below baseline or 90 mmHg and fluid resuscitation was ineffective, a continuous infusion of norepinephrine (0.03*kg/50 ml) was administered to maintain circulatory stability. If the SBP increased more than 30% above baseline, urapidil 5–12.5 mg was injected intravenously, with repeated doses if necessary. If an accidental conversion to open thoracotomy or significant bleeding occurred, the study was immediately stopped, and the patient was excluded.

### 2.4. Rescue measures

Postoperative resting pain with a VAS score > 4 was given an intramuscular tramadol hydrochloride injection of 0.1 g by the postoperative management physician, who did not know the group.

If the catheter was accidentally dislodged or removed due to complications within 72 h postoperatively in both groups, Patient-controlled intravenous analgesia (hydromorphone hydrochloride 0.08 mg/kg + flurbiprofen injection 3 mg/kg + tropisetron injection 5 mg + 0.9 saline to 200 ml) was given.

Preoperative ESPB block, intravenous nonsteroidal anti-inflammatory drugs, oral analgesics, and intravenous opioid rescue were prescribed and administered by anesthesiologists, following our hospital’s pain management protocol.

### 2.5. Pain evaluation

#### 2.5.1. Assessment of acute postoperative pain

Acute postoperative pain was systematically assessed at rest and during coughing at predefined time points (6, 12, 24, 48, and 72 h postoperatively) using the VAS. Specifically, the VAS is a scale with a 10 cm movable ruler, with 10 markings on one side, and ‘0’ and ‘10’ at each end, where ‘0’ represents no pain and ‘10’ represents the most severe pain that is intolerable. Based on the evaluation results, pain levels are classified into 4 grades: no pain (VAS = 0), mild pain (VAS 1–3), moderate pain (VAS 4–6), and severe pain (VAS 7–10). At our hospital, the 12 h postoperative period typically coincides with nursing shift changeovers, often occurring during night or early morning hours. This represents a high-risk period for breakthrough pain and inadequate analgesia. Assessing analgesic efficacy at this time point holds significant clinical practice implications. Furthermore, our clinical experience indicates that single-dose nerve blocks using ropivacaine without adjuvants typically provide analgesia lasting 6–12 h. We set the primary outcome at 12 h to capture the critical time window after the initial nerve block effect wanes, assessing whether the continuous infusion regimen can effectively sustain analgesia [17]. Additionally, we aim for patients to achieve their first ambulation and cough to clear secretions by 12 h postoperatively.

#### 2.5.2. Definition and assessment of CPSP

According to the International Pain Research Society [18], chronic pain in this study is defined as postoperative pain lasting for ≥3 months. During follow-up for chronic pain, study members contact the subjects 90 days after surgery by phone and specifically inquire whether they have experienced any persistent pain or discomfort at the surgical site (VATS incision), chest or back, axilla, or ipsilateral upper limb. If they indicate such pain or discomfort, they are asked to complete the Brief Pain Inventory Short Form Questionnaire (BPI-SF) and the Short Form McGill Pain Questionnaire (SF-15) to evaluate their nature and intensity. Finally, all subjects who meet the definition of CPSP and have an average BPI-SF pain intensity score of ≥1 or McGill (SF-15) score of ≥1 or both will be included in the data analysis. The McGill (SF-15) questionnaire [19] is a simplified version of the original McGill Pain Questionnaire, which consists of a Pain Severity Index that includes both sensory and emotional subscales. This questionnaire can be used to assess pain caused by neuropathic factors, with higher total scores indicating more severe pain experiences. The BPI-SF has been validated for assessing chronic pain [20], and it evaluates the location, severity, and impact of pain on patients’ quality of life, with impacts assessed through general activities, emotions, mobility, work, relationships with others, sleep, and life interests. The basic method involves using a 10 cm ruler with ‘0’ at one end representing no impact and ‘10’ at the other representing complete impact.

### 2.6. Anxiety state assessment

In this study, the State-Trait Anxiety Inventory (STAI) will be used to assess preoperative anxiety levels in patients. STAI is recognized as the gold standard for assessing preoperative anxiety [21] because it directly reflects the subjective feelings of anxious patients and effectively distinguishes between state and trait anxiety. The inventory includes 40 items, with 20 items for the state anxiety scale (STAI, Form Y-I, referred to as S-AI). Half of these items are negative emotional items, while the other half are positive emotional items. It is primarily used to evaluate immediate or specific time- or situation-specific fear, tension, anxiety, and nervousness or feelings. The 21st-40th items belong to the trait anxiety scale (STAI, Form Y-l, referred to as T-AI), which is used to evaluate regular emotional experiences. Among them, 11 items describe negative emotional items, while 9 items are positive emotional items. Each item has a specific scoring method: S-AI: 1 = none, 2 = some, 3 = moderate, 4 = very obvious; T-AI: 1 = almost no, 2 = some, 3 = often, 4 = almost always. Participants can choose the most appropriate score according to their own experience. Positive emotional items are scored in reverse order. Separate scores are calculated for the S-AI and T-AI scales, with a minimum of 20 and a maximum of 80, reflecting the level of state or trait anxiety.

The seven-item Generalized Anxiety Disorder Scale (GAD-7) mainly asks patients about changes in mental and emotional state after surgery [22]. Patients are asked to answer ‘none’, ‘sometimes’, ‘most of the time’, and ‘almost every day’ with scores of 0–3 points for symptoms such as feeling tense, anxious, angry; unable to stop, or control worrying; overwhelming concerns about many things; difficulty relaxing; feeling restless or easily irritable; and feeling afraid as if something terrible is going to happen. The higher the score, the more severe the anxiety. GAD-7 can be used as a screening tool for GAD conditions and can also be used to assess the severity of the condition based on the score: 0–4 points, normal levels; 5–9 points, mild anxiety; 10–13 points, moderate anxiety; 14–18 points, moderate to severe anxiety; and 19–21 points, severe anxiety.

### 2.7. Outcome measures

The primary outcome of this study is the resting VAS pain score at 12 h postoperatively. Concurrently, we measured and recorded the VAS pain score during coughing as an important secondary endpoint.

Secondary outcomes include the QoR-15 scale score at 12, 24, 48, and 72 h or discharge; the resting and coughing VAS pain score at 6, 24, 48, and 72 h postoperatively; the incidence of CPSP at 3 months postoperatively; the time to first postoperative analgesic requirement and the number of rescue analgesics used; the duration of regional block procedures and related complications (block failure or abnormalities, catheter displacement, blockage, leakage, infection, etc.); postoperative adverse events (nausea, vomiting, dizziness, urinary retention, skin pruritus, etc.); and levels of cytokines (TNF-α, IL-6, IL-8) in plasma preoperatively and at 48 h postoperatively.

Exploratory clinically relevant outcome measures include intraoperative haemodynamic parameters (mean arterial pressure, heart rate, SpO_2_, Bispectral Index) reflecting the quality of control over noxious stimuli during surgery, alongside overall satisfaction with analgesia to assess patients’ subjective experience (defined as: 1 = very satisfied, 2 = satisfied, 3 = neutral, 4 = dissatisfied).

### 2.8. Sample size calculation and statistical analyses

The sample size was calculated a priori based on the primary outcome: the resting VAS score at 12 h postoperatively. Data from our pilot study showed mean VAS scores of (3.0 ± 1.4) in the D + C-ESPB group and (4.1 ± 1.5) in the C-ESPB group, yielding a mean difference (δ) of 1.1 points and a pooled standard deviation (σ) of approximately 1.45. A difference of 1.1 points was considered clinically relevant, as it exceeds the established minimum clinically important difference (MCID) of 1.0 points for VAS in acute postoperative pain [23,24]. Using a two-sided independent *t* test with an alpha (α) level of 0.05 (*Z*_α_/2 = 1.96) and a power (1–β) of 90% (*Z*_β_ = 1.282), the required sample size per group was calculated using the following formula:

$$n=n=\frac{2{\left(z_{\alpha }+z^{\beta }\right)}^{2}*\sigma^{2}}{\delta^{2}}$$

Substituting the values: *n* = [2*(1.96 + 1.282)2*1.452]/1.12 ≈ 40.3. Therefore, a minimum of 41 patients per group was required. Accounting for a potential 15% dropout rate (e.g. due to loss to follow-up or catheter failure), we aimed to enroll 49 patients per group, resulting in a total sample size of 98. During the actual recruitment period, 90 patients were randomised, slightly below the initial target. However, the final data analysis demonstrated extremely high statistical power for the primary outcome.

Data entry and statistical analyses were conducted in a blinded fashion. The analysis was performed using SPSS 24.0 software (SPSS, Chicago, IL, USA). Parametricity of continuous variables was determined using the Shapiro–Wilk test. Normally distributed continuous variables are expressed as mean with SD, and non-parametric variables as median with quartiles (P_25_, P_75_). Unpaired *t* test and Mann–Whitney *U* test were used to compare normally and non-normally distributed data, respectively, between the two study arms. Kruskal–Wallis rank sum test for multiple comparisons. Fisher’s exact test was used for comparing categorical data. The repeated-measures ANOVA was used for analysing the VAS pain scores (both resting and coughing) and QoR-15 scores across all time points. We have also detailed the process for handling violations of sphericity using the Greenhouse–Geisser correction, and explained that post hoc simple effects analyses with Bonferroni correction were conducted only when a significant interaction effect was found. A *p* value of <0.05 was considered statistically significant.

## 3. Results

**Figure 1.**
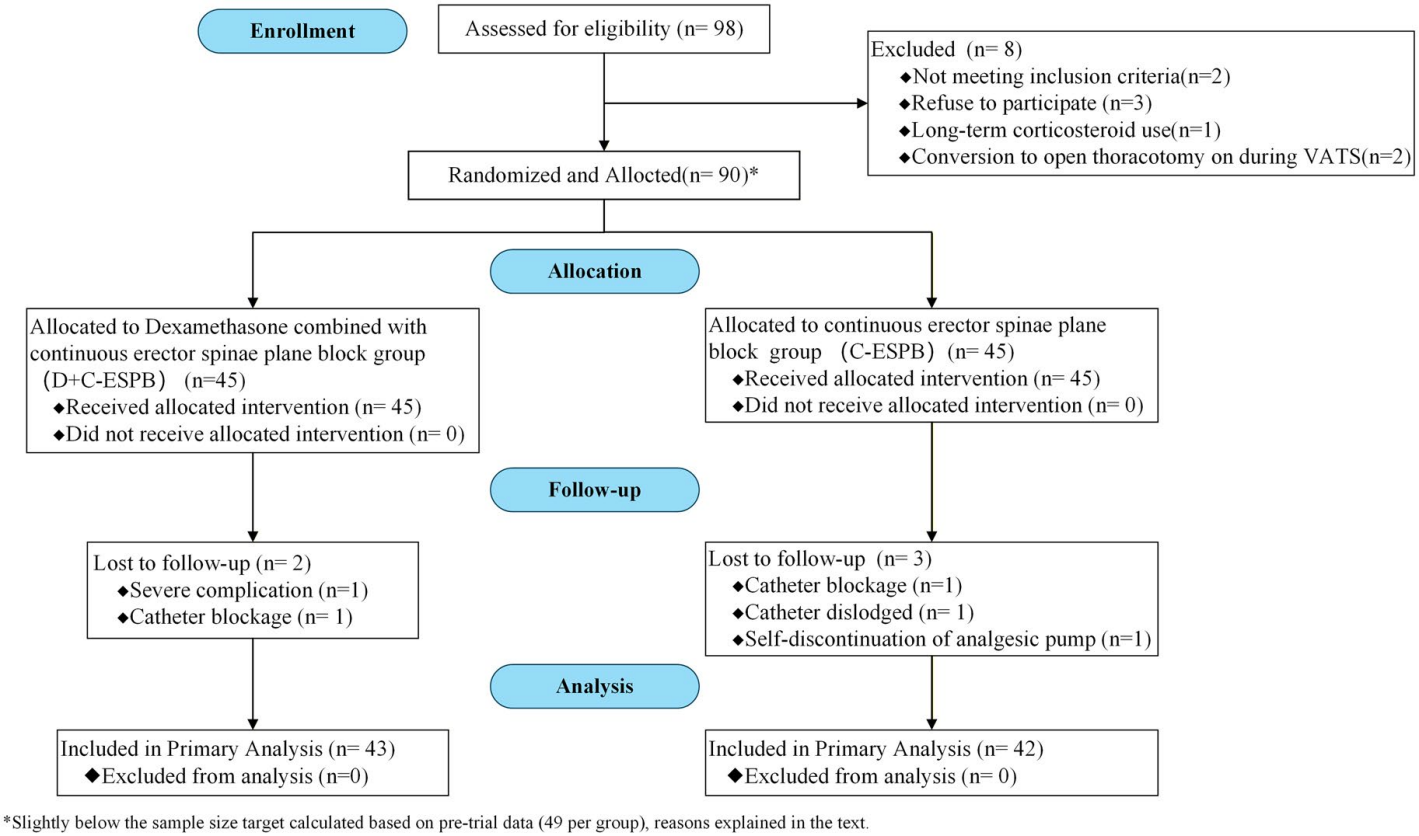
Consolidated standards of reporting trials (CONSORT) flowchart. D + C-ESPB, Dexamethasone + Continuous Erector Spinae Plane Block; C-ESPB, Continuous Erector Spinae Plane Block.

Figure 1 illustrates the Consolidated Standards of Reporting Trials (CONSORT) flow diagram. Between December 10, 2022, and August 30, 2023, a total of 90 eligible patients were enrolled, with 45 in the D + C-ESPB group and 45 in the C-ESPB group. Two patients in the D + C-ESPB group were excluded from the final analysis (surgery-related complications, *n* = 1; catheter occlusion, *n* = 1). Three patients in the C-ESPB group were also excluded (catheter occlusion, *n* = 1; catheter dislodgement, *n* = 1; and Self-discontinuation of the analgesic pump, *n* = 1). Therefore, all 90 randomised participants were included in the intention-to-treat analysis, but due to loss to follow-up, the per-protocol analysis comprised 85 participants. We analyzed 43 subjects in the D + C-ESPB group and 42 subjects in the C-ESPB group. Exclusion of subjects in whom we failed to achieve a block catheter was not specified a priori. The analysis was effective per protocol analysis.

### 3.1. Baseline data

The preoperative demographics of both groups were similar, including age, height, weight, BMI, ASA, educational level, type of surgery, duration of surgery, anesthesia time, blockage range, and preoperative QoR-15 scores. Anesthetic and surgical techniques used were also similar between the two groups (Table 1).

**Table 1.** Demographics and baseline characteristics.

	D + C-ESPB group	C-ESPB group	*p* value
	(*n* = 43)	(*n* = 42)	
Age (y) Mean (SD)	49.19 (10.71)	54.24 (8.93)	0.349
**Sex**			
Female [*n* (%)]	31 (58.1%)	28 (54.8%)	0.754
Male [*n* (%)]	22 (41.9%)	24 (45.2%)	
Weight (kg) Mean (SD)	62.76 (9.96)	62.34 (11.14)	0.354
Height (cm) Mean (SD)	162.53 (7.75)	1161.76 (8.76)	0.29
BMI (kg/m^2^) Mean (SD)	23.68 (2.70)	23.70 (2.82)	0.869
**ASA physical status [***n* (%)**]**			
I	0	0	1
II	41 (95.3%)	41 (97.6%)	
III	2 (4.7%)	1 (2.4%)	
**Education level**			
Junior Secondary and below [*n* (%)]	16 (37.2%)	23 (54.8%)	0.104
Junior Secondary and above [*n* (%)]	27 (62.8%)	19 (45.2%)	
Preoperative QoR-15 scale score Median (*P*_25_,*P*_75_)	146 (142,148)	146 (143,148)	0.757
**Type of surgery [***n* (%)**]**			
Lobectomy	4 (9.3%)	5 (11.9%)	0.814
Cuneatectomy	22 (51.2%)	23 (54.8%)	
segmental lung resection	17 (39.5%)	14 (33.3%)	
Duration of surgery (min) Median (*P*_25_, *P*_75_)	65 (55,85)	75 (55,101.25)	0.292
Duration of anesthesia (min) Median (*P*_25_, *P*_75_)	99 (85,127)	105 (136.25)	0.463
**Blockage range (*n*)**			
T_2_–T_6_	0	1	1*
T_2_–T_7_	2	2	1^#^
T_2_–T_8_	9	6	0.422^&^
T_2_–T_9_	6	6	0.965^&^
T_3_–T_6_	3	1	0.625^#^
T_3_–T_7_	6	7	0.728^&^
T_3_–T_8_	12	12	0.946^&^
T_3_–T_9_	2	1	1^#^
T_4_–T_6_	0	1	1*
T_4_–T_7_	0	1	1*
T_4_–T_10_	1	0	0.494*
T_5_–T_7_	0	1	1*
Undetectable^ф^	2	3	0.978^#^

D + C-ESPB, Dexamethasone + Continuous Erector Spinae Plane Block； C-ESPB, Continuous Erector Spinae Plane Block； ASA, American Society of Anesthesiologists； QoR-15, Quality of recovery-15; SD, Standard deviation.

*Fisher’s exact probability method was applied.

^#^A corrected chi-square test was applied.

^&^Pearson’s chi-squared test was applied.

^ф^Blocking range not measured within 30 min.

### 3.2. Primary outcome

**Figure 2.**
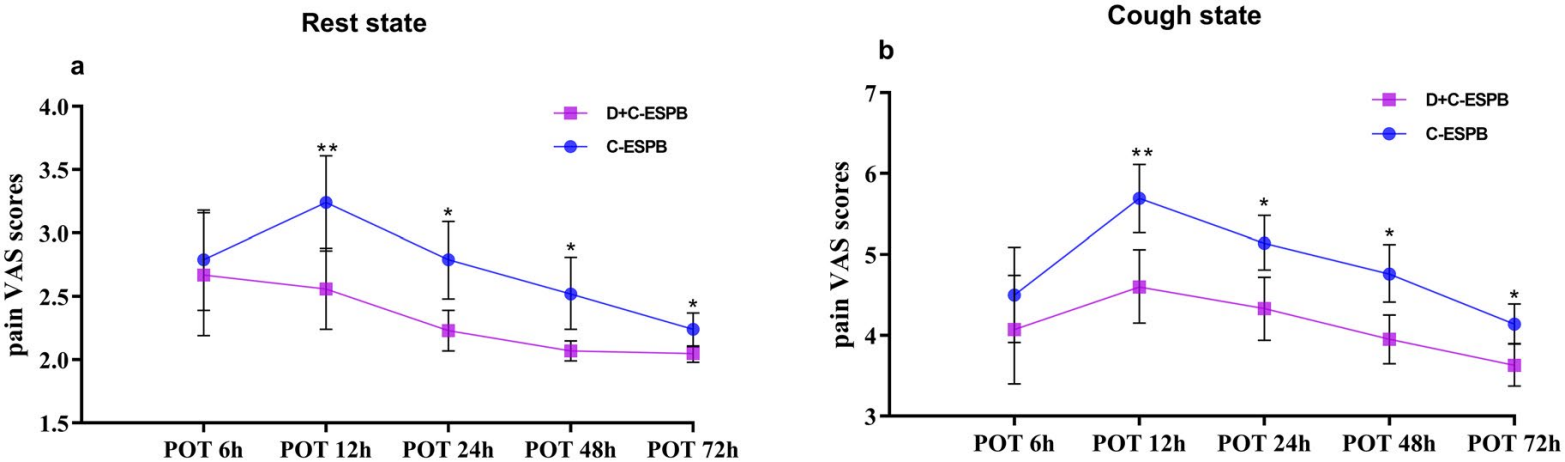
Pain VAS scores tendencies by groups at rest state (a) and cough state (b) postoperatively. ‘*’, *p* < 0.05 vs. C-ESPB; ‘**’, *p* < 0.001 vs C-ESPB; POT, Postopertive time, D + C-ESPB, Dexamethasone + Continuous Erector Spinae Plane Block; C-ESPB, Continuous Erector Spinae Plane Block.

A repeated-measures analysis of variance was performed on resting pain VAS scores across the 72 h postoperative period. The data violated the sphericity assumption (Mauchly’s *W* = 0.376, *p* < 0.001), and results are therefore reported with the Greenhouse-Geisser correction. This revealed a statistically significant main effect of group (*F* = 11.143, *p* = 0.001, partial η^2^ = 0.118), with the D + C-ESPB group exhibiting lower pain scores than the C-ESPB group. A significant main effect of time was also observed (*F* = 10.844, *p* < 0.001, partial η^2^ = 0.116). No significant group-by-time interaction was detected (*F* = 1.653, *p* = 0.183, partial η^2^ = 0.020). For the predefined primary endpoint, the resting pain VAS score at 12 h postoperatively was lower in the D + C-ESPB group [2.56 (1.03) vs 3.24 (1.21); mean difference −0.680, 95% CI, (−1.17, −0.19), *p* = 0.006, Cohen’s *d* = 0.61] (Figure 2).

### 3.3. Secondary outcomes

#### 3.3.1. Post-operative pain

Following repeated-measures ANOVA, post hoc comparisons with Bonferroni correction were performed. Resting pain VAS scores were significantly lower in the D + C-ESPB group relative to the C-ESPB group at 24 h [2.23 (0.53) vs 2.79 (0.98), mean difference 0.553, *p* = 0.002], 48 h [2.07 (0.26) vs 2.52 (0.92), mean difference 0.454, *p* = 0.002], and 72 h [2.05 (0.21) vs 2.24 (0.43), mean difference 0.192, *p* = 0.011]. Statistically significant within-group differences over time were identified in the C-ESPB group [e.g.,12 h vs 48 h [3.24 (1.21) vs 2.52 (0.92), mean difference 0.714, *p* = 0.002], 12 h vs 72 h [3.24 (1.21) vs 2.24 (0.43), mean difference 1.000, *p* < 0.001], as well as between 24 h vs 72 h [2.79 (0.98) vs 2.24 (0.43), mean difference 1.000, *p* < 0.001]. In contrast, no significant within-group differences between consecutive time points were observed in the D + C-ESPB group (Figure 2).

Analysis of coughing pain VAS scores (with Greenhouse–Geisser correction) identified significant main effects for group (*F* = 16.128, *p* < 0.001, partial η^2^ = 0.163) and time (*F* = 13.378, *p* < 0.001, partial η^2^ = 0.139), but no significant interaction (*F* = 1.009, *p* = 0.377). Post hoc tests (Bonferroni-corrected) confirmed significantly lower scores in the D + C-ESPB group at 12 h [4.60 (1.48) vs 5.69 (1.35), mean difference 1.068, *p* < 0.001], 24 h [4.33 (1.27) vs 5.14 (1.07), mean difference 0.817, *p* = 0.002], 48 h [3.95 (0.97) vs 4.76 (1.14), mean difference 0.808, *p* < 0.001], and 72 h [3.63 (0.85) vs 4.14 (0.78), mean difference 0.515, *p* = 0.005]. The C-ESPB group showed multiple within-group differences (e.g. 12 h vs 72 h [5.69 (1.35) vs 4.14 (0.78), mean difference 1.548, *p* < 0.001], while the D + C-ESPB group exhibited fewer variations 12 h vs 72 h [4.60 (1.48) vs 3.63 (0.85), mean difference 0.977, *p* < 0.001]; 24 h vs 72 h [4.33 (1.27) vs 3.63 (0.85), mean difference 0.698, *p* < 0.001] (Figure 2). 9 patients in the D + C-ESPB group required rescue analgesia, compared to twenty-three in the C-ESPB group (*p* = 0.004, Bonferroni correction). The total number of rescue analgesic treatments administered was 15 and 32, respectively (*p* = 0.68) (Table 2). Postoperative tramadol consumption over 72 h was lower in the D + C-ESPB group [0 (0,100) vs 100 (75,100), *z* = –3.807, *p* < 0.001].

**Table 2.** Analgesic treatment within 72 h in both groups.

	D + C-ESPB group	C-ESPB group	Median difference	*Z*/χ^2^	*P* value
	(*n* = 43)	(*n* = 42)	(95% CI)		
Rescue treatment^a^ (*n*)	9	23		10.36	0.002
Total number of rescue analgesia (bpm)	15	32		7.372	0.008
Number of resuscitation analgesia at 12 h postoperatively (bpm)	8	15		0.17	0.68
Time to first resuscitation analgesia (h)	7.46(5.36,18.09)	8.67 (4.75,10.16)	1.25 (−0.17,7.68)	0.922	0.363
The first compression time of the pump (h)	13.36 (4.92,21.56)	8.66 (4.50,15.14)	2.11 (−2.58,10)	1.03	0.301
Total number of effective compressions	1 (1,6)	4 (3,7)	−2 (−4,0)	2.369	0.018
Total number of compressions	4 (1,3)	9 (5,12.5)	−3 (−7,1)	1.311	0.19
Analgesic pump consumption (ml)	241 (204,292)	268 (233.75,300)	−13.5 (−36,1)	1.509	0.131
Tramadol consumption	0 (0,100)	100 (75,100)	0 (0,0)	−3.807	<0.001

D + C-ESPB, Dexamethasone + continuous erector spinae plane block; C-ESPB, Continuous erector spinae plane block.

^a^ Tramadol hydrochloride injection was used to relieve pain.

#### 3.3.2. QoR-15

**Figure 3.**
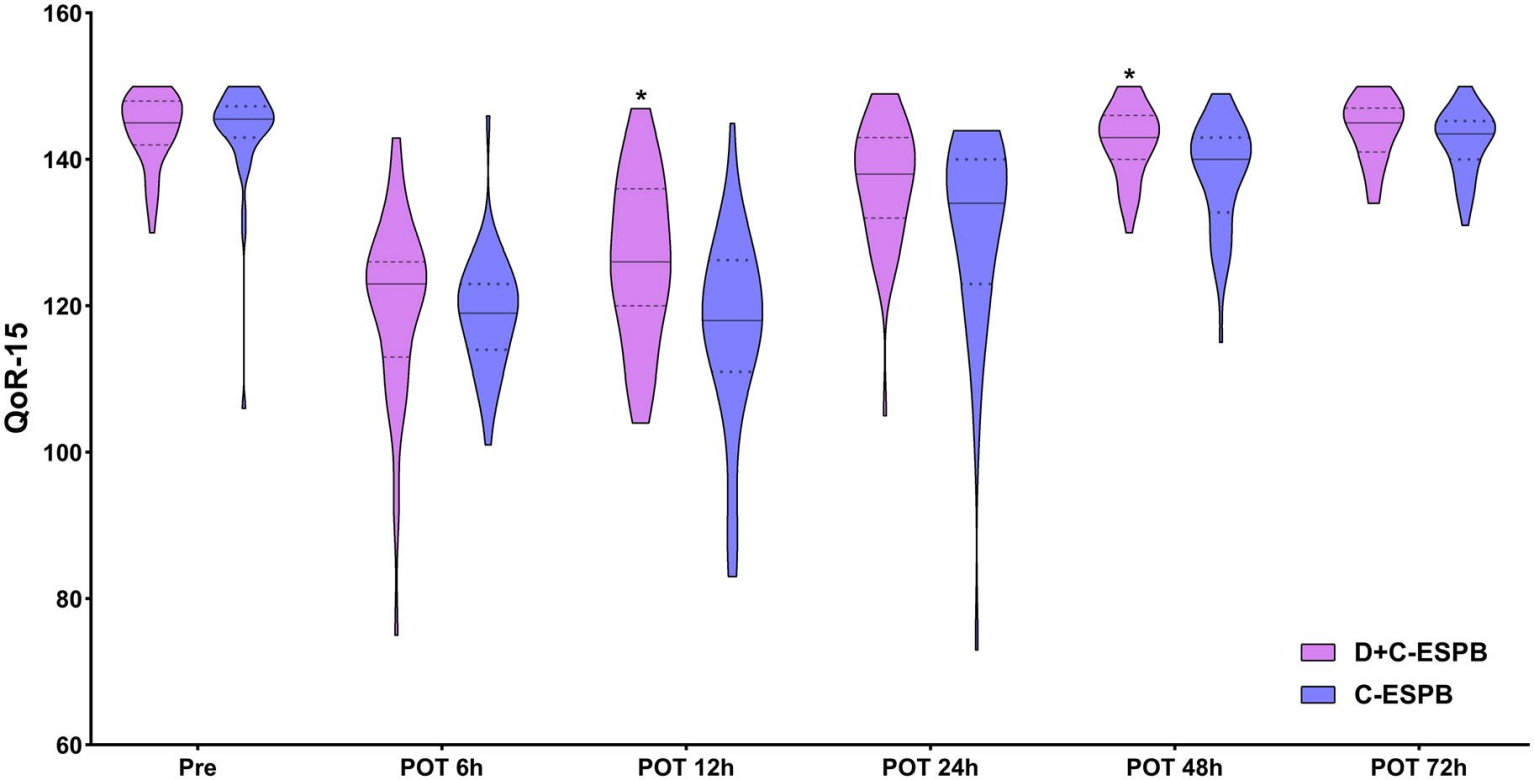
Quality of recovery (QoR-15) at different time points postoperatively. *p < 0.05 vs C-ESPB; ‘–’ and ‘…’ denote median and 25% and 75% inter-quartile range, respectively; D + C-ESPB, dexamethasone-complexed ropivacaine continuous erector spinae planar block; C-ESPB, continuous erector spinae planar block; Pre, preoperative; POT, postoperative time; QoR-15, 15-item quality of recovery scale.

**Figure 4.**
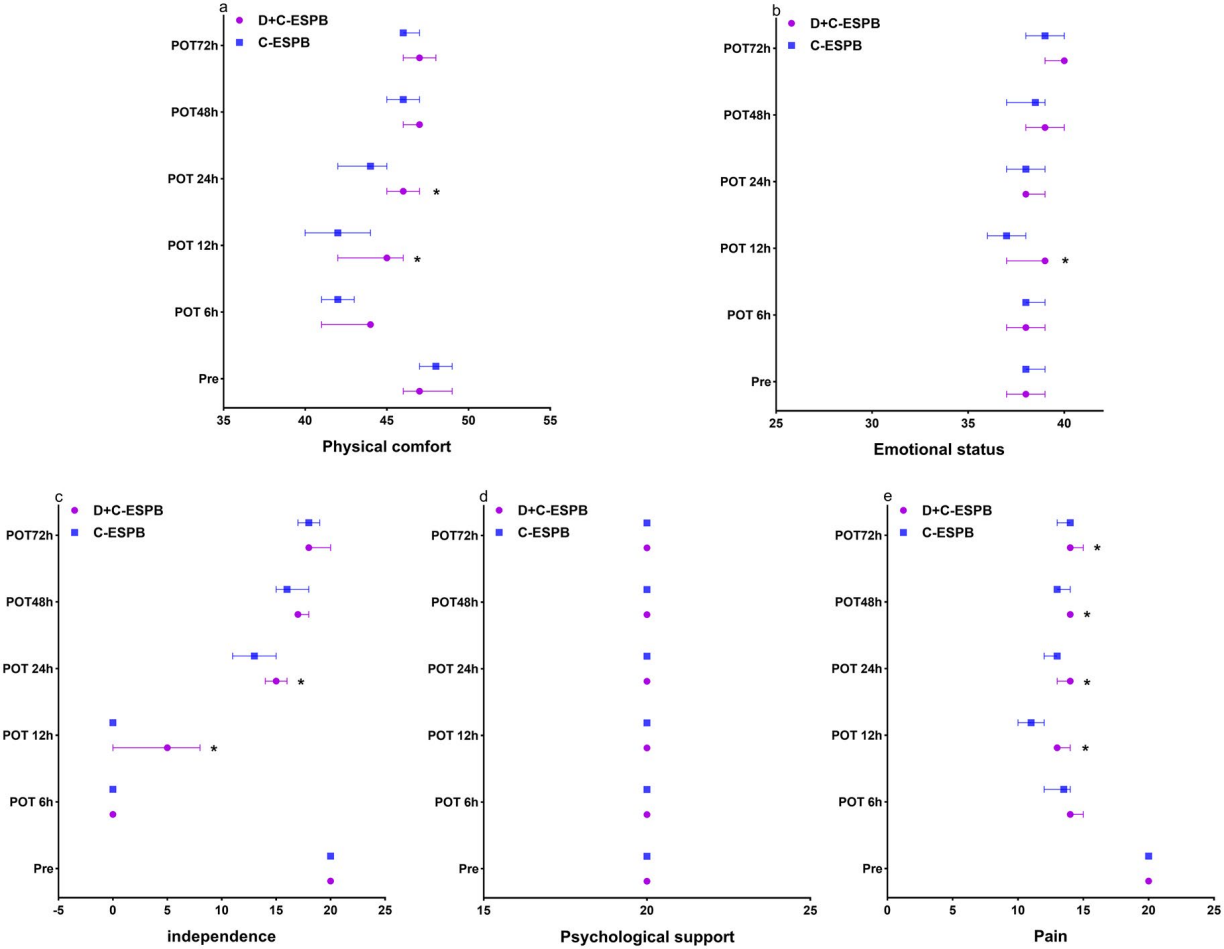
Difference in median QoR-15 domains by groups at physical comfort (a), emotional state (b), self-care ability (c), psychological support (d), and pain (e) postoperatively. *p < 0.05 vs C-ESPB; error bars represent 95% asymptotic HodgeseLehmann confidence limits; D + C-ESPB, dexamethasone combined with ropivacaine continuous erector spinae plane block; C-ESPB, continuous erector spinae plane block; Pre, preoperative; POT, postoperative time; QoR-15, 15-item quality of recovery scale.

Changes in QoR-15 scores over time were evaluated with repeated-measures ANOVA. Following a violation of sphericity (Mauchly’s *W* = 0.301, *p* < 0.001), results are reported with Greenhouse–Geisser corrections. No statistically significant main effect of group was detected (*F* = 2.848, *p* = 0.095, partial η^2^ = 0.033). Statistically significant main effects were identified for time (*F* = 192.207, *p* < 0.001, partial η^2^ = 0.698) and for the group-by-time interaction (*F* = 4.028, *p* = 0.004, partial η^2^ = 0.046). Subsequent post hoc analysis with Bonferroni correction revealed that QoR-15 scores were significantly higher in the D + C-ESPB group compared to the C-ESPB group at the 12 h [124.70 (12.48) vs 117.26 (12.24); mean difference −7.436, *p* = 0.007], 48 h [141.60 (5.51) vs 138.98 (6.64); mean difference −2.628, *p* = 0.050] (Figure 3). Within-group comparisons showed that the C-ESPB group had significant differences between pre and POT6h (mean difference 24.881, *p* < 0.001), POT12h (27.048, *p* < 0.001), POT24h (12.643, *p* < 0.001), and POT48h (5.333, *p* < 0.001), but not between pre and POT72h (1.714, *p* = 1.000). The D + C-ESPB group exhibited significant differences between pre and POT6h (25.930, *p* < 0.001), POT12h (18.977, *p* < 0.001), and POT24h (8.395, *p* < 0.001), but not between pre and POT48h (2.070, *p* = 1.000) or POT72h (−0.512, *p* = 1.000). Analysis of QoR-15 subdomains revealed significantly better scores for the D + C-ESPB group in physical comfort [45 (41, 46) vs 42 (38, 44.25), *p* = 0.009], physical independence [5 (0, 10) vs 0 (0, 4.25), *p* = 0.01], and emotional state [39 (37, 39) vs 37 (35, 38), *p* = 0.006] at 12 h postoperatively, and in physical comfort [46 (42, 47) vs 44 (39, 46), *p* = 0.007] and physical independence [15 (13, 17) vs 13 (10, 16), *p* = 0.033] at 24 h postoperatively (Figure 4).

#### 3.3.3. CPSP

At the 3-month postoperative assessment, 80 participants were included in the CPSP analysis (two non-respondents were excluded from the original 85). The incidence of CPSP was 32.5% (*n* = 27/80), with a higher prevalence in females (74.1%). Two patients were excluded from the analysis for non-respondents (*n* = 2). At the time of the telephone interview (3 months postoperatively), among subjects with CPSP, the median with quartiles (*P*_25_, *P*_75_) BPI ‘average’ pain severity score among subjects who reported any CPSP was 2 (1,3), the BPI pain interference composite score was 0 (0,0), and the SF-15 score was 0 (0,1). No significant difference in CPSP incidence was found between the D + C-ESPB (31%, 13/42) and C-ESPB groups (34%, 14/41) (*p* = 0.756). Among those with CPSP, there were no significant between-group differences in BPI ‘average’ pain severity [2 (1,3) vs 2 (1.5,2.5) (*p* = 0.959)] and SF-15 scores [0 (0,1) vs 0 (0,1.5) (*p* = 0.667)]. Preoperative State-Trait Anxiety Inventory (STAI) scores were higher in patients who developed CPSP compared to those who did not [72.74 (12.36) vs 52.39 (8.55), *p* < 0.001] (Table 3). Interestingly, in all 27 patients who developed CPSP at 3 months postoperatively, the 12 h postoperative QoR-15 was less than 121, with a significantly higher incidence in the C-ESPB group than in the D + C-ESPB group [*n* (%),14 (100%) vs. 11 (74.6%), *p* = 0.22].

**Table 3.** Comparison of preoperative state-Trait anxiety inventory and CPSP between the two groups of patients [mean (SD)].

Items	T-AI	S-AI	Positive mood	Negative mood	Overall score
CPSP group(*n* = 27)	35.00 (6.66)	37.74 (7.21)	42.78 (7.92)	29.96 (6.99)	72.74 (12.37)
N-CPSP group(*n* = 56)	26.09 (4.44)	26.55 (4.73)	28.16 (7.18)	24.48 (4.47)	52.64 (8.56)
Mean difference (95%CI)	8.91 (6.46,11.36)	11.19 (8.56,13.82)	14.2 (11.16,18.08)	5.48 (2.49,8.46)	20.10 (15.46,24.73)
*t*	7.241	8.459	8.4	3.725	8.63
*p* value	<0.001	<0.001	<0.001	0.001	<0.001

CPSP, chronic persistent surgical pain; T-AI, trait anxiety scale; S-AI, state anxiety scale.

#### 3.3.4. Organ recovery

**Figure 5.**
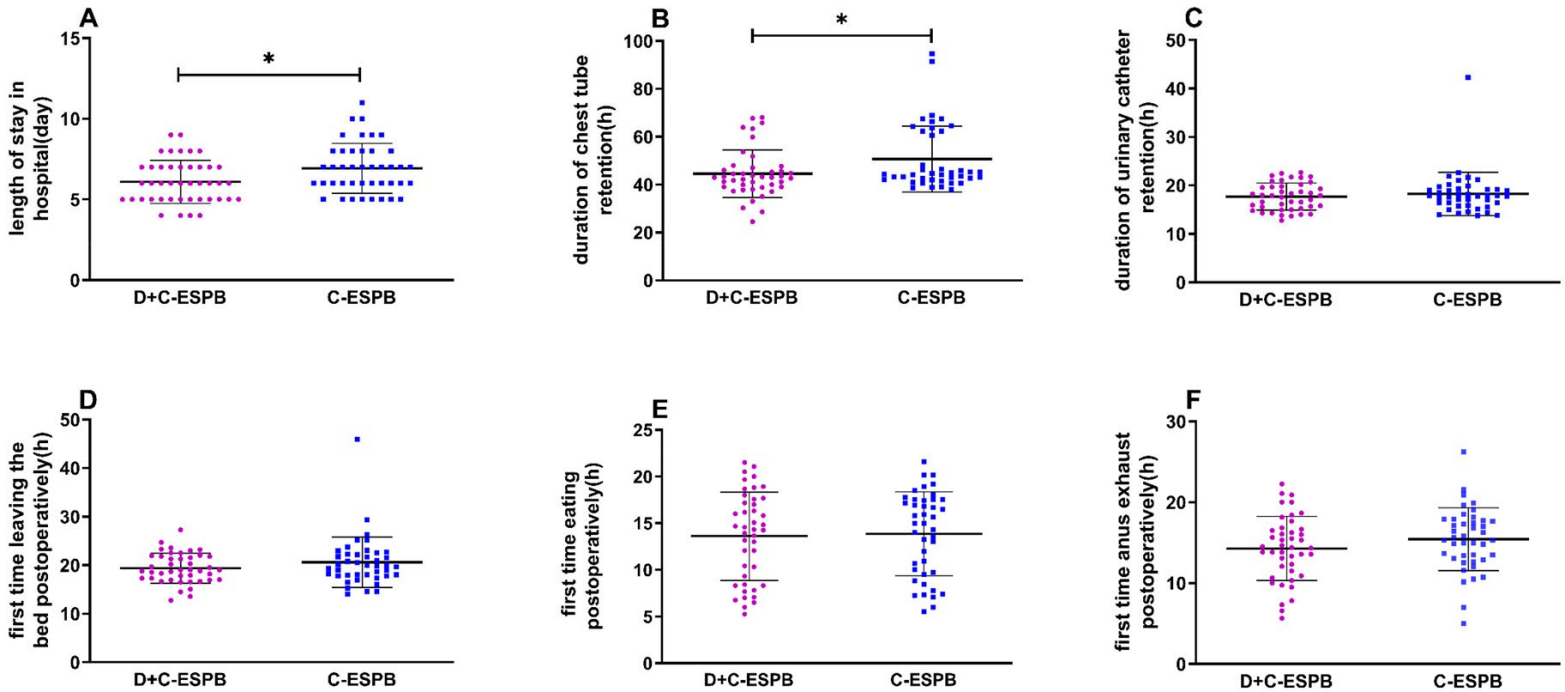
Scatter plot analysis of length of stay in hospital (A), duration of chest tube retention (B), duration of urinary catheter retention (C), and first time leaving the bed postoperatively(D), first time eating postoperatively (E), and first time anus exhaust postoperatively (F) in the D + C-ESPB and C-ESPB groups. The differences between groups were significant for length of stay in hospital (*p* = 0.01) and duration of chest tube retention (*p* = 0.023). D + C-ESPB, Dexamethasone + Continuous Erector Spinae Plane Block; C-ESPB, Continuous Erector Spinae Plane Block; POT, Postoperative time.

Compared to the C-ESPB group, the D + C-ESPB group showed a reduction in hospitalization length [6.09 (1.34) vs 6.93 (1.55) days, *p* = 0.01] and chest tube indwelling time [44.57 (9.94) vs 50.68 (13.79)h, *p* < 0.001]. While patients in the D + C-ESPB group experienced shorter postoperative ambulation, first-time eating, first flatus time, and urinary catheter indwelling time compared to those in the C-ESPB group, the differences between the two groups were not statistically significant (Figure 5).

#### 3.3.5. Stress reaction

At 48 h postoperatively, the lymphocyte count was lower in the D + C-ESPB group compared to the C-ESPB group [mean (SD), 10.35 (4.48) vs. 8.37 (3.57), *p* = 0.027]. No other statistically significant between-group differences were observed for IL-6, IL-8, TNF-α, WBC count, or neutrophil count. Within-group comparisons revealed significant changes from preoperative baseline at 48 h. Both groups showed significant changes in IL-6, WBC, neutrophil, and lymphocyte counts. In the D + C-ESPB group, blood glucose [median (P25, P75): 7.5 (6.4, 9.00) vs. 6.1 (5.4, 7.50), *p* < 0.001] and lactic acid [1.09 (0.8, 1.3) vs. 0.9 (0.7, 1.2), *p* < 0.001] were increased. In the C-ESPB group, TNF-α levels were decreased [1.63 (1.14, 2.18) vs. 6.85 (5.35, 8.88), *p* = 0.003].

#### 3.3.6. Adverse events

There was no statistically significant difference between the two groups in terms of intraoperative opioid (sufentanil, remifentanil, etc.) consumption as well as postoperative PACU stay, anesthesia recovery, and hospitalization cost (*p* > 0.05, Table 4). Additionally, no significant differences (*p* > 0.05) were observed between the two groups regarding catheter-related complications (dislodgement, infection, blockage, etc.), pulmonary complications (pneumothorax, lung atelectasis, pneumonia, etc.), and anesthesia-related complications (nausea, vomiting, itchy skin, dizziness, constipation, etc.) (Table 4).

**Table 4.** Secondary outcomes included intra-operative opioid, vasoactive drugs, satisfaction with analgesic pumps in 72 h, regional anesthesia outcomes, adverse events, pulmonary complications and post-operative indicators after minimally invasive thoracic surgery between the D + C-ESPB group and the C-ESPB group; all values shown are mean (SD), median (*P*_25_, *P*_75_), or [*n* (%)]; no statistical adjustment was made for multiple comparisons.

Items	D + C-ESPB group	C-ESPB group	Difference and 95% CI (%)	*t*/*z*/χ^2^	*p* value
	**(*n* = 43)**	**(*n* = 42)**			
***Intra-operative opioid and vasoactive drugs***					
Norepinephrine dosage (µg) Median (*P*_25_, *P*_75_)	120 (71,157.5)	100 (78,130)	4 (−20,40)	0.567	0.571
Intra-operative remifentanil dosage (µg) mean (SD)	227.4 (100.83)	226.9 (81.9)	0.47 (−43.1, 44)	0.021	0.277
Intra-operative sufentanil dosage (µg) median (*P*_25_, *P*_75_)	35 (35,40)	35 (35,40)	0 (0,5)	0.757	0.449
Fluid volume (ml) mean (SD)	1232.61 (276.37)	1247.88 (248.70)	11.24 (−103.02,125.5)	0.039	0.845
Blood loss (ml) median (*P*_25_,*P*_75_)	100 (100,150)	100 (100,150)	0 (0,0)	−0.1	0.921
***Post-operative indicators***					
PACU length of stay (min) median (*P*_25_, *P*_75_)	77.40 (28.36)	93.90 (39.72)	−10 (−25,0)	2.67	0.102
Anesthesia recovery time (min) median (*P*_25_, *P*_75_)	22.23 (15.50)	33.95 (23.6)	−5 (−11,0)	2.461	0.117
Tracheal tube extraction time (min) median (*P*_25_, *P*_75_)	52.46 (22.26)	61.98 (33.89)	−5 (−17,5)	0.828	0.363
Total cost of hospitalisation ($) median (*P*_25_, *P*_75_)	6767.14 (5813.69,7317.43)	6777.59 (5685.43,8223.32)	−2442.93 (−6855.55,1969.68)	4.76	0.53
***Analgesia satisfaction***					
**POT 6 h**					
Very satisfied [*n* (%)]	29 (67.4%)	15 (35.7%)	3.729 (1.520,9.146)	8.566	0.003
Satisfied [*n* (%)]	8 (18.6%)	18 (42.9%)	0.305 (0.114,0.813)	5.886	0.015
Generally satisfied [*n* (%)]	5 (11.6%)	9 (21.4%)	0.482 (0.147,1,584)	1.483	0.023
Dissatisfied [*n* (%)]	1 (2.4%)	0	–	–	1*
**POT 12 h**					
Very satisfied [*n* (%)]	26 (60.5%)	8 (19%)	6.5 (2.431,17.376)	15.186	<0.001
Satisfied [*n* (%)]	15 (34.9)	12 (28.6)	1.339 (0.535,3.552)	0.391	0.532
Generally satisfied [*n* (%)]	2 (4.7%)	18 (42.9%)	0.065 (0.014,0.305)	17.237	<0.001
Dissatisfied [*n* (%)]	0	4 (9.5%)	–	2.436	0.119^#^
**POT24**					
Very satisfied [*n* (%)]	25 (58.1%)	8 (19%)	5.903 (2.216,15.726)	13.671	<0.001
Satisfied [*n* (%)]	15 (34.9%)	19 (45.2%)	0.648 (0.271,1.553)	0.949	0.33
Generally satisfied [*n* (%)]	3 (7%)	14 (33.3%)	0.096 (0.025,0.378)	14.154	<0.001
Dissatisfied [*n* (%)]	0	1 (2.4%)	–	–	0.494*
**POT48**					
Very satisfied [*n* (%)]	25 (58.1%)	5 (11.9%)	10.278 (3.376,31.286)	19.888	<0.001
Satisfied [*n* (%)]	16 (37.2%)	26 (61.9%)	0.365 (0.152,0.877)	5.184	0.023
Generally satisfied [*n* (%)]	2 (4.7%)	10 (23.8%)	0.156 (0.032,0.763)	6.432	0.011
Dissatisfied [*n* (%)]	0	1 (2.4%)			0.494*
**POT72**					
Very satisfied [*n* (%)]	25 (58.1%)	7 (16.7%)	6.944 (2.522,19.119)	15.568	<0.001
Satisfied [*n* (%)]	17 (58.1%)	33 (78.6%)	0.178 (0.068,0465)	13.367	<0.001
Generally satisfied [*n* (%)]	1 (58.1%)	2 (4.8%)	0.476 (0.042,5.459)		0.983^#^
Dissatisfied [*n* (%)]	0	0	–	–	–
***Regional anesthesia outcomes***					
Time to insert regional catheter (min) Median (*P*_25_, *P*_75_)	8 (6,10)	7 (5.75,8)			0.259
Depth to insert regional catheter (cm) Median (*P*_25,_ *P*_75_)	6 (6,7)	6 (5,7)			0.715
Duration of regional catheter stay (h) Median (*P*_25_, *P*_75_)	65 (56,70)	66.5 (62.75,70)			0.543
Catheters dislodged <24 h [*n* (%)]	0	0			
Catheter intentionally removed <24 h [*n* (%)]	0	0			
Catheters intact at 24 h [*n* (%)]	43 (100%)	42 (100%)			
Catheter dislodged at >24 h but < 48 h [*n* (%)]	0	0			
Catheter intentionally removed at >24 h but <48 h [*n* (%)]	0	0			
Catheter intact at 48 h [*n* (%)]	43 (100%)	42 (100%)			
Catheter intentionally removed at >48 h but <72 h [*n* (%)]	0	0			
Catheter intact at 72 h [*n* (%)]	43 (100%)	42 (100%)			
Catheter blockage [*n* (%)]	0	0			
Block complication [*n* (%)]	2 (4.65%)	2 (4.76%)			
	Puncture site pain	Puncture site pain			
***Adverse events***					
Nausea [*n* (%)]	2 (4.65%)	2 (4.76%)			1
Vomiting [*n* (%)]	2 (4.65%)	2 (4.76%)			1
Itchy skin [*n* (%)]	0	1 (2.38%)			0.494
Dizziness [*n* (%)]	0	1 (2.38%)			0.494
Constipation [*n* (%)]	0	0			
***Pulmonary complications***					
Pneumothorax [*n* (%)]	18 (41.86%)	25 (59.52%)		2.652	0.103
Atelectasis [*n* (%)]	2 (4.65%)	5 (11.9%)		0.675	0.411
Pneumonia [*n* (%)]	1 (2.33%)	3 (7.14%)		0.288	0.592

D + C-ESPB, Dexamethasone + Continuous Erector Spinae Plane Block; C-ESPB, Continuous Erector Spinae Plane Block; POT, Postoperative time; PACU, Postanesthesia care unit; SD, standard deviation.

*Fisher’s exact probability method was used.

^#^A corrected chi-square test was used.

### 3.4. Exploratory outcomes

#### 3.4.1. Hemodynamics

In exploratory analysis, we found that there were no statistically significant differences (*p* > 0.05) between the MAP, SPO2, BIS, and HR of the two groups of patients in the preoperative period (Pre), the immediate moment of induction of anesthesia (T0), skin incision (T1), entry into the thorax (T2), single-lung ventilation for 30 min (T3), flushing of the thorax (T4), the end of single-lung ventilation (T5), and the end of the operation (T6).

#### 3.4.2. Satisfaction

Significant differences in patient satisfaction with analgesia were observed between the groups across all postoperative assessments. Satisfaction was higher in the D + C-ESPB group. The proportions of patients in each satisfaction category differed significantly: very satisfied (130/215 vs 43/210, difference and 95% CI 4.508 (2.963, 6.859), *p* < 0.001); satisfied (71/215 vs 108/210, difference and 95% CI 0.466 (0.315, 0.689), *p* < 0.001); generally satisfied (13/215 vs 53/210, difference and 95% CI 0.191 (0.100, 0.362), *p* < 0.001) The difference in the number of dissatisfied patients (C-ESPB vs D + C-ESPB: 6 vs 1) was not statistically significant (*p* = 0.12) (Table 4).

## 4. Discussion

Determining the best approach for pain relief after VATS remains a key clinical issue, particularly when it comes to managing moderate-to-severe pain and supporting quicker recovery post-surgery. The research shows that incorporating dexamethasone as an adjuvant to a continuous ropivacaine infusion for ESPB notably enhances early postoperative pain relief in patients undergoing VATS for pulmonary nodule removal. The main outcome, resting pain at 12 h, was notably reduced in the D + C-ESPB group, with an average difference of −0.68 points (*p* = 0.006), indicating a medium-to-large effect size (Cohen’s *d* = 0.61). The reduction is approaching the established benchmark for the minimal clinically significant difference in VAS pain scores, underlining its clinical importance [24,25]. These pain management benefits were associated with a marked decrease in the consumption of rescue opioids (tramadol) and correlated with better recovery quality at designated postoperative times.

For VATS, the ESPB has emerged as an effective element of multimodal analgesia, with a more advantageous risk-benefit profile than thoracic epidural or paravertebral techniques [26–28]. Unlike studies investigating single-shot ESPB for various thoracic procedures[29,30], our research utilized a continuous catheter method to extend and possibly improve pain relief. Although dexamethasone is a recognized adjuvant for lengthening the duration of single-injection peripheral nerve blocks, its impact on continuous local anesthetic infusions remains unclear. Our findings suggest that its primary role in a continuous catheter regimen may extend beyond merely prolonging duration—which is the principal goal in single-shot blocks—to enhancing the density and reliability of the evolving block over time, as evidenced by the sustained reductions in both resting and dynamic pain scores.

Our results, which show early enhancement of pain relief, are in line with the general idea of using perineural adjuvants, but they should be viewed within the specific context of continuous blockade. Unlike earlier research that examined single-shot ESPB with dexamethasone for different thoracic operations, our study concentrated solely on continuous ESPB for the removal of pulmonary nodules [31–33]. This methodological distinction is crucial. The main purpose of dexamethasone in a single-injection scenario is to increase the limited time the local anesthetic is effective. Its use in a continuous infusion setting is conceptually distinct and less frequently discussed, to possibly move towards enhancing the quality and uniformity of the sustained blockade. The marked decrease in rescue analgesia incidents and tramadol use in our D + C-ESPB group backs the theory that dexamethasone aids in establishing a more effective and dependable continuous block. The comparable pain scores at the 6 h point between the groups probably indicate the strong impact of the initial surgical dose of ropivacaine given to all patients, aimed at creating a basic block [34]. The way perineural dexamethasone works involves multiple factors and seems to be mainly local. Growing evidence indicates that its analgesic potentiation is mediated through local genomic and non-genomic actions on neuronal membranes, rather than systemic anti-inflammatory effects [12,35,36]. Consistent with this paradigm, we observed no consistent between-group differences in systemic inflammatory markers (IL-6, IL-8, TNF-α), arguing against a major systemic anti-inflammatory mechanism for the analgesic benefit. The isolated finding of a lower lymphocyte count in the D + C-ESPB group at 48 h is pharmacologically plausible, reflecting the known capacity of dexamethasone to induce lymphocyte redistribution [37], but should not be overinterpreted as the primary analgesic mechanism.

We assessed patient-reported recovery using the QoR-15 scale. The D + C-ESPB group reported higher scores at 12 and 48 h postoperatively, a finding consistent with recent publications [38]. The between-group difference at 12 h (7.4 points) exceeded the established minimal clinically important difference of 6.0 points [39], indicating a patient-perceivable improvement in early recovery quality in the intervention group. However, the absence of a significant main effect for ‘group’ in the repeated-measures analysis tempers this conclusion, suggesting the adjuvant’s influence is most pronounced in the immediate postoperative phase. While this multimodal analgesic regimen provided an early advantage in domains such as physical comfort and independence at 12–24 h, its impact on the global recovery trajectory over 72 h appears more modulated. The uniformly low incidence of postoperative nausea and vomiting (≈4.7%) highlights the efficacy of our structured multimodal prophylactic strategy, which included intraoperative dexamethasone and a 5-HT_3_ antagonist, thereby confounding the assessment of any specific antiemetic contribution from perineural dexamethasone [40].

This study also observed positive trends in several objective clinical metrics in the D + C-ESPB group. The duration of chest tube drainage was significantly shorter in the D + C-ESPB group compared to the control group [44.57 (9.94) vs 50.68 (13.79) hours, *p* < 0.001]. This is likely attributable to superior analgesia, as effective pain control facilitates earlier and more productive coughing and deep breathing, enabling patients to meet clinical criteria for chest tube removal sooner [41]. This advantage may subsequently contribute to the observed reduction in hospital length of stay [6.09 (1.34) vs 6.93 (1.55) days, *p* = 0.01]. Although the length of stay is influenced by numerous non-analgesic factors, optimised pain management is unequivocally a cornerstone of ERAS [42]. Moreover, aligning with these favorable clinical advancements, patients in the D + C-ESPB group expressed notably greater satisfaction with the pain management plan at every postoperative time point (Table 4). Satisfaction, centered on the patient, is an outcome that encompasses the efficacy of pain control, the profile of side effects, and overall comfort [38]. Although satisfaction was an exploratory outcome in this study, the higher satisfaction scores, coupled with reduced rescue analgesia requirements and better early recovery quality scores, collectively sketch a promising picture of the multifaceted benefits afforded by dexamethasone as an adjuvant.

An important secondary aim of this research was to examine how dexamethasone-enhanced continuous ESPB might affect CPSP. The 3-month CPSP incidence rate of 32.5% is consistent with the ranges reported for thoracic surgery [43,44]. Although a numerical reduction was observed in the D + C-ESPB group (31% vs. 34%), this difference was not statistically significant. This finding is congruent with the complex, multifactorial aetiology of CPSP, where a single perioperative intervention may have a limited impact on long-term prevalence. Notably, our findings support known risk factors, showing that a larger percentage of CPSP cases are found in females (74.1%) [45]. Furthermore, we observed that all patients who developed CPSP had a QoR-15 score below 121 at 12 h postoperatively, irrespective of group allocation. This aligns with previous work linking poorer early recovery quality to a higher risk of CPSP [2] and suggests that early postoperative recovery status may be a more potent predictor of chronic pain than the specific regional analgesic or multimodal technique employed in this study.

Several limitations of this work warrant acknowledgement. Initially, the study was carried out at one location with a sample size designed to assess the main pain outcome, rather than secondary outcomes like CPSP incidence or inflammatory markers. Secondly, while the patient-reported outcome (QoR-15) we used is validated, its subjective nature remains a limitation, albeit supported by objective data such as opioid consumption and rescue events. Thirdly, postoperative monitoring of catheter position and block extent was not continuous, possibly leading to the limited catheter-related issues observed. Fourthly, the optimal concentration of dexamethasone in a continuous infusion remains undefined and merits further investigation. Finally, the follow-up durations for catheter usage and CPSP evaluation were restricted to 72 h and 3 months, respectively; further research over extended periods is necessary.

To sum up, incorporating dexamethasone into a continuous ropivacaine ESPB infusion greatly improves early postoperative pain relief and decreases the need for opioids following VATS for the removal of pulmonary nodules—with an effect size indicating clinical relevance—but also improves patient-perceived recovery quality in the immediate postoperative period. Nonetheless, it did not have a significant impact on systemic inflammatory markers or the occurrence of CPSP after 3 months. This research demonstrates the potential and pain-relieving effectiveness of incorporating adjuvants in continuous regional analgesia catheters, an approach that is not widely studied and may aim to enhance block quality instead of just prolonging its duration.

## 4. Conclusion

In VATS patients, perineural dexamethasone alongside continuous ESPB enhances early pain management, recovery quality, and opioid-sparing, but does not decrease the occurrence of CPSP at 3 months. Further investigations need to verify these outcomes in large-scale multicenter studies and examine ideal dosing strategies and possible synergies with non-pharmacological techniques.

## Ethics approval and consent to participate

Ethical approval for this study was obtained from the Ethics Committee of the Army Medical University Xinqiao Hospital (ID: 2022-Research No. 293-01; June 15, 2022). Written informed consent was acquired from all participants. The trial was registered with the China Clinical Trial Registry before commencement (Registration No. ChiCTR2200066531; Registration Date: December 8, 2022; URL: http://www.chictr.org.cn/listbycreater.aspx), ensuring compliance with international standards, such as the Declaration of Helsinki. All procedures performed in this study were in accordance with the ethical standards of the responsible committee on human experimentation. Informed consent was obtained from all participants or their legal representatives for their inclusion in the study. For any clinical data collection, participants were fully informed of the objectives, methods, and potential impacts of the study. Participants were also made aware of their right to withdraw from the study at any time without consequence. The consent process ensured that participants understood their data might be used for publication in an anonymized format, and no identifiable patient information would be disclosed. Copies of the ethics approval and signed consent forms are available upon request from the editorial team or regulatory bodies. The study adheres to all applicable ethical guidelines, including those about patient confidentiality and data protection. If any additional documentation is needed, I am prepared to provide it upon request.

## Supplementary Material

CONSORT 2010 Checklist.doc

## Data Availability

The datasets generated and analyzed during the current study are available from the corresponding author on reasonable request.
